# Supplementary figures and images for: Entorhinal transformations in abstract frames of reference
Source: PLoS Biol. 2019 May 2;17(5):e3000230. doi: 10.1371/journal.pbio.3000230 (PMC6497227; doi:10.1371/journal.pbio.3000230)

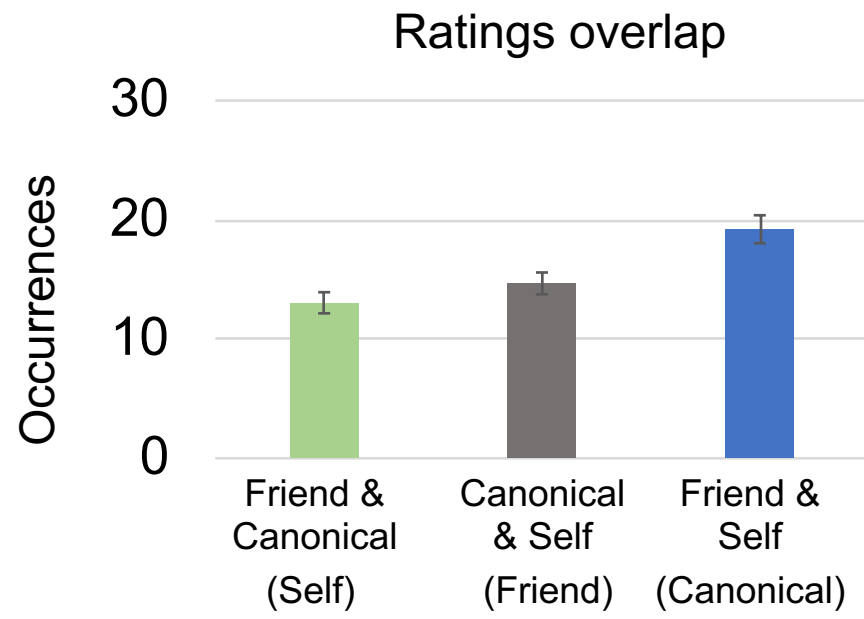

Supplement: S1 Fig — Significant effect of condition for ratings overlap (the same ratings) between the two individuals (P < 0.001). Individuals being compared are listed below each bar with the corresponding anchor/condition name listed in parentheses. Occurrences are out of the 100 trials per condition. See S4 Data for subject data. (PDF) [file pbio.3000230.s001.pdf]

### Choice Discrimination

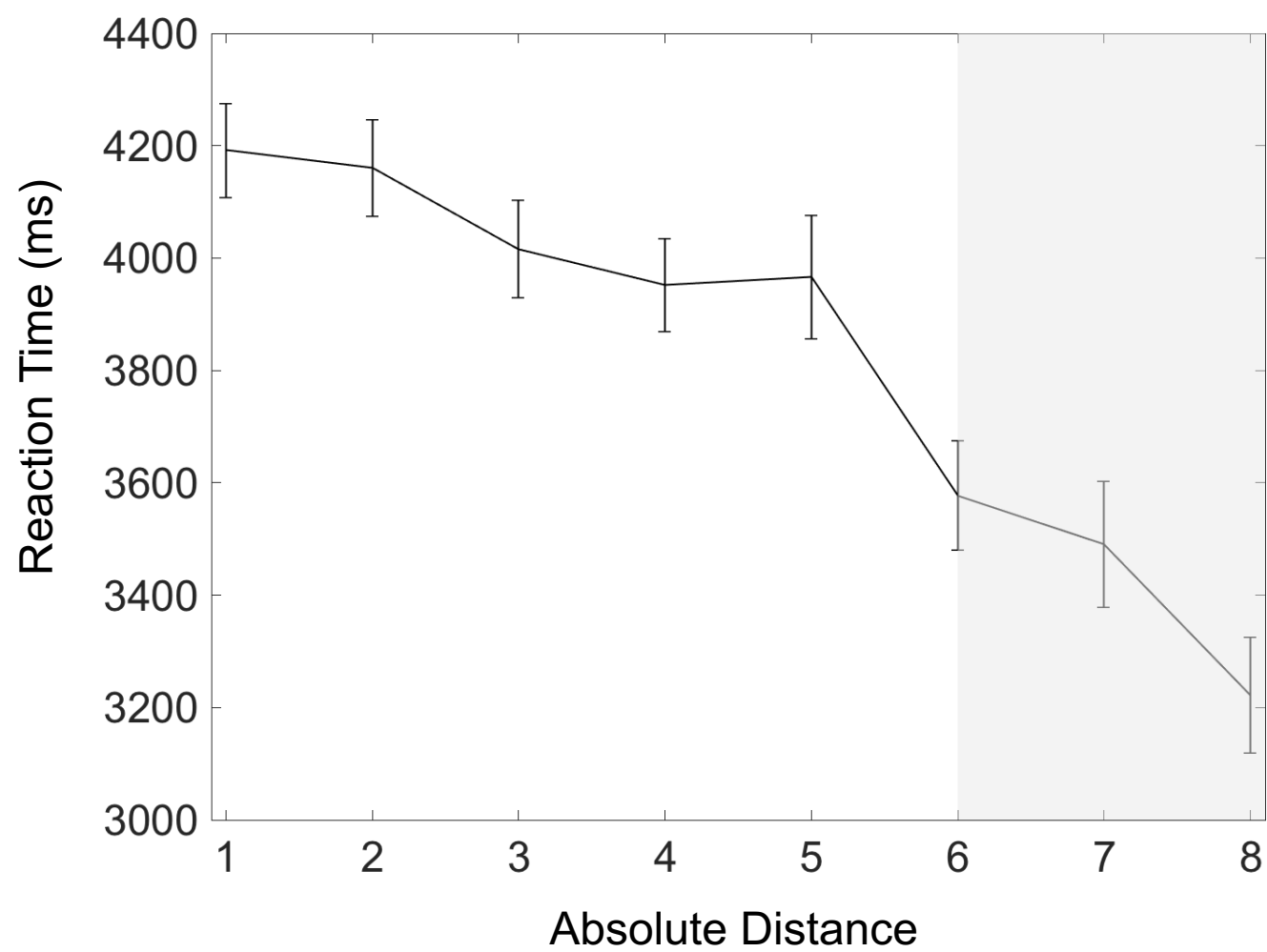

Supplement: S2 Fig — Significant relationship (P < 0.001) between decision speed (reaction time) and the absolute distance between strangers’ ratings and the nonanchor individuals for each trial. The absolute value of absolute distances were rounded to the closest integer and plotted from 1 to 8. Seven and 8 on the x-axis are shaded in gray, because only 18/24 and 13/24 subjects had trials with absolute distances of 7 and 8, respectively. Error bars showing mean ± SEM. See S5 Data for subject data. (PDF) [file pbio.3000230.s002.pdf]

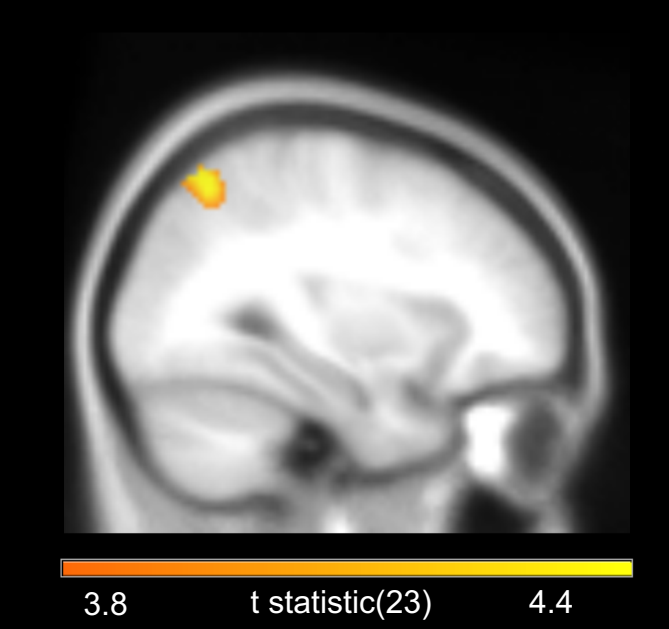

Supplement: S3 Fig — Sagittal image showing superior parietal lobule effect of mentally rescaling anchor towards the periphery in either direction, as seen in Fig 1D. Highlighted region survived cluster-level FWE correction at P < 0.05 and image is displayed at an uncorrected statistical threshold of P < 0.001. FWE, family-wise error (PDF) [file pbio.3000230.s003.pdf]

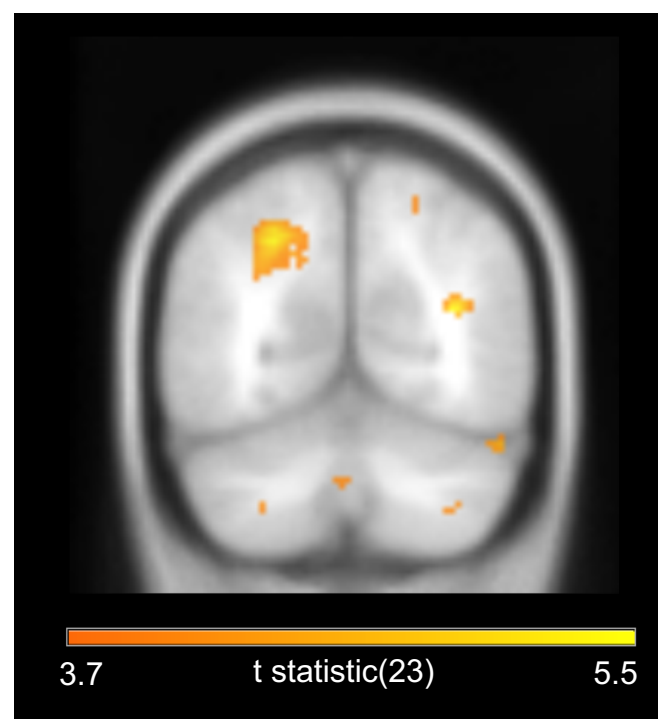

Supplement: S4 Fig — Coronal image of increased intraparietal sulcus activity for correct versus incorrect choices. Highlighted region survived cluster-level FWE correction at P < 0.05 and image is displayed at an uncorrected statistical threshold of P < 0.001. FWE, family-wise error. (PDF) [file pbio.3000230.s004.pdf]

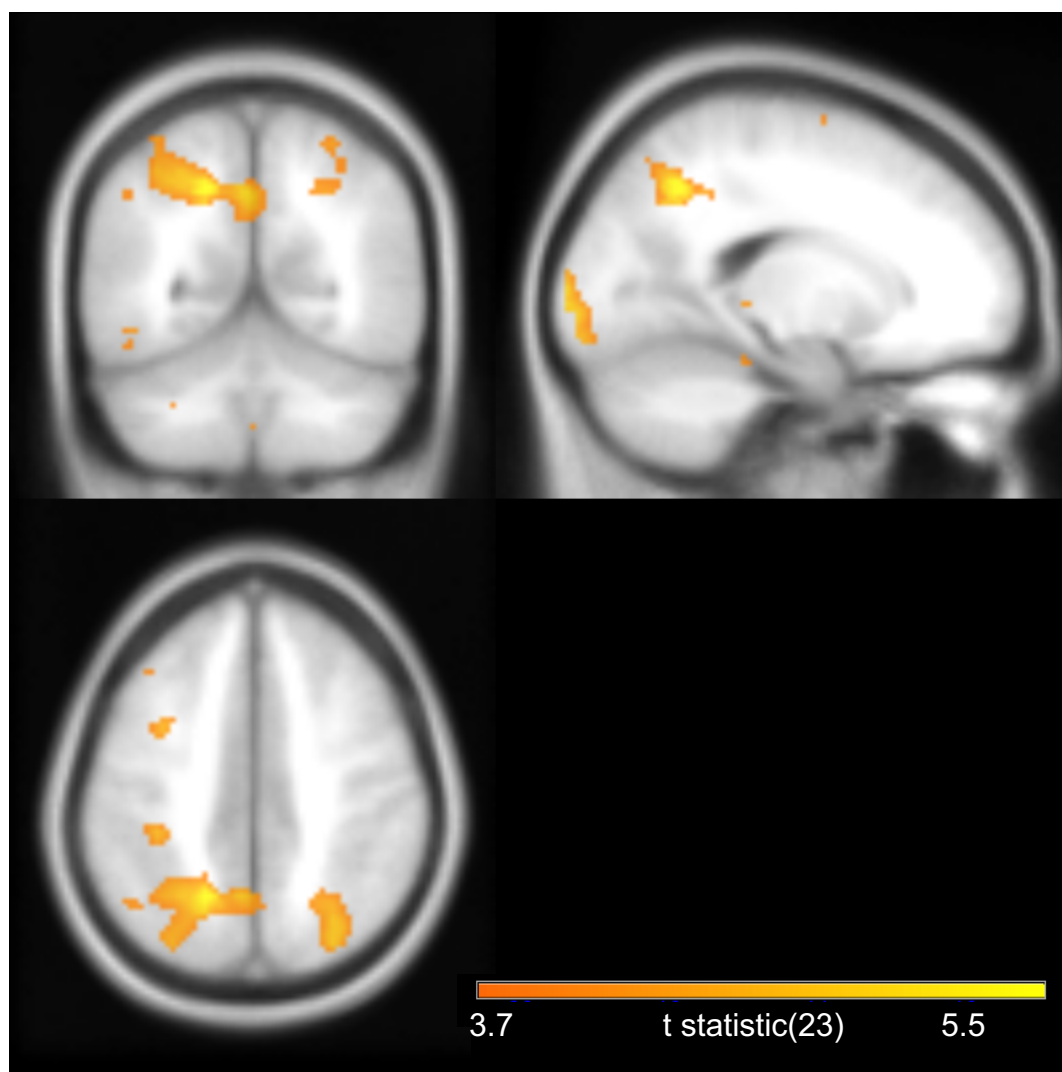

Supplement: S5 Fig — Images of increased posterior parietal cortex and precuneus activity for slower RTs. Highlighted region survived cluster-level FWE correction at P < 0.05 and image is displayed at an uncorrected statistical threshold of P < 0.001. RT, reaction time. (PDF) [file pbio.3000230.s005.pdf]
