# Supplementary material for: Entorhinal transformations in abstract frames of reference
Source: PLoS Biol. 2019 May 2;17(5):e3000230. doi: 10.1371/journal.pbio.3000230 (PMC6497227; doi:10.1371/journal.pbio.3000230)
Supplement: S1 Table — Last 10 were used during practice trials and first 100 were used for the fMRI task. fMRI, functional magnetic resonance imaging. (DOCX) [file pbio.3000230.s006.docx]

1. Sending a message using mostly emojis 2. Eating spicy food 3. Following a TV series 4. Falling asleep in the car 5. Cycling 6. Meeting friends at the pub 7. Eating fast food 8. Downloading a movie 9. Speaking on the phone in a foreign language 10. Eating sushi 11. Wearing boots 12. Reading a novel 13. Having a house party 14. Growing vegetables 15. Taking an evening class 16. Going to a nightclub 17. Wearing a suit jacket 18. Sending a postcard when on holiday 19. Eating chocolate 20. Drinking coffee in the morning 21. Taking a dance class 22. Joining a political march 23. Volunteering 24. Jogging 5 miles 25. Smoking a cigarette 26. Needing caffeine to wake up in the morning 27. Catching a taxi 28. Travelling abroad for work 29. Listening to music on their way to work 30. Looking at new cars 31. Going to the drycleaners 32. Watching cartoons 33. Fixing things around the house 34. Reading a newspaper (online or print) 35. Having a meeting with a co-worker of a different nationality 36. Eating a kebab 37. Wearing a leather jacket 38. Hiring a person to clean their house 39. Hosting a dinner for friends 40. Visiting a children’s museum 41. Sending a message via SMS 42. Dying hair 43. Painting their home 44. Leave a voicemail after a call 45. Drinking tea 46. Visiting an art museum 47. Communicate via email 48. Chat via instant messaging 49. Speak via video chatting 50. Having a conversation lasting more than an hour 51. Speaking with family 52. Ordering take away pizza 53. Cooking dinner 54. Making lunch 55. Going out to dinner with friends. 56. Visiting a library 57. Making a new post on a blog 58. Sharing gossip 59. Joining a book club 60. Following politics 61. Sun tanning 62. Playing a board game 63. Watching a romantic movie 64. Holidaying in a tropical location 65. Going to a concert 66. Waking up early during the week 67. Attending a musical 68. Watching sport on TV 69. Playing videogames 70. Wearing a hat 71. Going on a road trip 72. Driving to work 73. Taking more than an hour to get ready in the morning 74. Wearing a sleeveless shirt 75. Walking to work 76. Going to a comedy show 77. Riding the bus 78. Spending a weekend afternoon on the sofa 79. Buying clothes from a secondhand shop 80. Buying designer clothes 81. Owns a pair of running shoes 82. Buying a new piece of furniture 83. Buying things from a moving sale 84. Decorating their place 85. Going to an antiques store 86. Hanging out with their neighbours. 87. Going to a yoga class 88. Spending a holiday lying down on the beach 89. Going for a hike 90. Sleeping more than 7 hours a night 91. Playing football with friends 92. Meditating 93. Doing sudoku 94. Taking a nap 95. Staying up late during the week 96. Falling asleep in front of the television 97. Sleeping through their alarm 98. Sleeping on a flight 99. Taking the train 100. Going to the airport 101. Using a motorbike 102. Going to a work conference 103. Going out for lunch during the week 104. Speaking multiple languages at work 105. Working from home 106. Supervising other people 107.Changing jobs 108. Receiving a work-related phone call. 109. Checking work-related email at home 110. Taking a coffee break at work
